# Does hematology rotation impact the interest of internal medicine residents in considering hematology as a career?

**DOI:** 10.1186/s12909-024-05192-w

**Published:** 2024-03-02

**Authors:** Mamoun Hassan Sharief, Assem A. Elghazaly, Abdullah Mohammad Al Abbas, Raed Saleh Al Basri, Samy A. Alsirafy

**Affiliations:** 1 Department of Adult Haematology-Oncology, King Saud Medical City, Riyadh, Saudi Arabia; 2 Palliative Medicine Unit, Department of Clinical Oncology and Nuclear Medicine, Kasr Al-Ainy Faculty of Medicine, Cairo University, Cairo, 11562 Egypt

**Keywords:** Internal medicine, Residents, Subspecialty rotation, Hematology, Career

## Abstract

**Background:**

The ongoing need for hematologists is not met in many parts of the world. The hematology rotation during internal medicine residency is an opportunity to attract more physicians to the hematology field. This study aimed to assess the impact of a hematology rotation on internal medicine residents’ interest in considering a hematology career.

**Methods:**

Internal medicine residents were invited to complete an anonymous questionnaire before and after a mandatory hematology rotation. Their interest in pursuing a hematology career was assessed by asking them to rate “Consider hematology as a career” on a 0 to 10 scale (0 = never, 10 = strongly agree). In addition, viewing the hematology workload as manageable, comfort in dealing with cancer and satisfaction with the hematologist lifestyle were assessed before and after the rotation.

**Results:**

Sixty out of 62 IM residents completed the pre- and post-hematology rotation questionnaire (response rate 97%). 80% were in the age range of 25–29 years and 73% were males. Two-thirds were in the senior level (3rd and 4th year) of their residency program and 40% had a prior rotation in a hematology unit. Rating considering hematology as a career increased significantly from a median of 7 (IQR: 5–9) pre-rotation to 8.5 (IQR: 7–10) post-rotation (p = 0.0018). Subgroup analysis showed a significant increase in interest among subgroups except residents > 29 years of age, those with prior hematology rotation and junior residents (1st and 2nd year residency). The change in viewing hematology workload as manageable, comfort in dealing with cancer patients and perceiving the hematologist lifestyle as satisfactory were strongly positively correlated with the change in considering hematology as a career (*p* = 0.0014, < 0.0001 and < 0.0001; respectively).

**Conclusions:**

A hematology rotation is associated with an increase in the interest of internal medicine residents in considering hematology as a career. Further research is needed to Identify factors that may make hematology rotations an effective tool in attracting residents to the hematology field.

## Introduction

There is a growing unmet demand for hematologists worldwide [1–4]. The local situation is not an exception as the number hematologists in Saudi Arabia is 4 per million people [5]. Although this ratio is better than in lower- and middle-income countries where the number of hematology specialists is less than 1 per million people [2] it is significantly lower than in western high-income countries like Canada where the ratio is 13 per million people [6]. The shortage in hematologists calls for finding strategies that make more physicians specialize in hematology.

Different subspecialty training is known to have variable effects and outcomes. This is attributed to multiple factors including the setting of training, workload, knowledge gained, psychological stress, involvement in research and physician lifestyle [7–12].

Exposure to medical branches during graduate training is one of the strong factors that impact the interest of trainees in pursuing specialties [13]. As per the National Training Authority of Saudi Arabia, Internal Medicine residents must go through at least 8 weeks of mandatory Clinical Hematology rotation and the management of both malignant and non-malignant hematological conditions is part of the internal medicine curriculum [14]. This hematology rotation represents an opportunity to attract physicians in training to the hematology field. However, the contrary may happen, and a specialty rotation may have a negative impact on the interest. A study found that an inpatient hematology-oncology rotation is associated with a decreased interest in the oncology career [8].

The aim of this study was to determine the impact of hematology rotation on the interest of internal medicine residents in considering hematology as a career and possible factors that may impact their interest.

## Methods

This prospective observational study was conducted in the period from December 2019 to May 2021 at King Saud Medical City (KSMC), Riyadh, Saudi Arabia.

### Participants and setting

Participants were internal medicine residents from different institutions performing their hematology rotations at the hematology unit of KSMC. Residents who performed a prior hematology rotation were not excluded.

The Saudi board program of internal medicine consists of four years of full-time residency training in internal medicine and its branches. It is divided into two levels: junior level (R1 – R2) and senior (R3 – R4), each consisting of 2 years of training. It is mandatory for internal medicine residents to go through an at least 8-week clinical hematology rotation during residency. This can be either as an 8-week one block or been divided into shorter periods.

The hematology unit is part of the Hemato-Oncology Department of KSMC which is a 1250-bed central tertiary care hospital. The unit is responsible for the investigation and management of adult patients with both benign and malignant hematological disorders. The service is provided through an inpatient unit, consultation team, outpatient clinics, and on-call emergency duty.

### The survey and other data collected

Before and after the hematology rotation residents were asked to complete an anonymous questionnaire in which they rate on a 0 to 10 scale the following statements regarding hematology specialty: “Consider hematology as a career” (0 = never, 10 = strongly agree), “Manageable workload” (0 = intolerable, 10 = very manageable), “Comfort in dealing with cancer patients” (0 = totally uncomfortable, 10 = very comfortable), and “Perception of hematologist lifestyle” (0 = totally unsatisfactory, 10 = very satisfactory). In addition, in the post-rotation questionnaire, the residents were asked to rate on a 0 to 10 scale teaching/training by hematology staff (0 = unsatisfactory, 10 = very satisfactory), gaining knowledge in general hematology (anemia, hemoglobinopathies, bone marrow failure,.) hematological malignancies, bleeding and thrombosis, and emergencies (0 = none, 10 = excellent), and usefulness in preparation for the internal medicine board exam (0 = not useful at all, 10 = very useful).

The following data were also collected: age, gender, internal medicine training center, level of training, prior rotation in hematology, and its level, type, and duration.

### Statistical analysis

Categorical data were described as numbers and percentages and continuous data as mean with standard deviation (SD) or median with interquartile range (IQR) as appropriate. The normal distribution of continuous variables was tested using the Shapiro-Wilk test. The Wilcoxon test for paired samples was used to compare the pre- and post-rotation 0–10 scale ratings. The correlation between considering hematology as a career and other ratings was tested using Spearman’s rank correlation test. A p-value < 0.05 was considered significant. Statistical tests were performed using MedCalc® Statistical Software version 22.009 (MedCalc Software Ltd, Ostend, Belgium).

## Results

Of 62 IM residents, 60 completed the pre- and post-rotation questionnaires during their hematology rotation at KSMC (response rate: 96.8%). Their characteristics are illustrated in Table 1. The majority (80%) were in the age range of 25–29 years and 73% were males. Almost two-thirds were in a senior level of training and the internal medicine training center was KSMC in 75%. 40% of residents had a prior hematology rotation, mainly (50%) as a part of their internal medicine residency training.

**Table 1:** Characteristics of 60 internal medicine residents

	n (%)
**Age** (years)	
25–29	48 (80)
30–34	9 (15)
> 34	3 (5)
**Gender**	
Male	44 (73.3)
Female	16 (26.7)
**Internal medicine training center**	
King Saud Medical City	45 (75)
King Salman Hospital	11 (18.3)
Other	4 (6.7)
**Training level**	
R1	3 (5)
R2	19 (31.7)
R3	23 (38.3)
R4	15 (25)
**Prior hematology rotation**	
No	36 (60)
Yes	24 (40)
**Prior hematology rotation level** (*n* = 26)	
Intern	2 (8.3)
Medical student	9 (37.5)
Non-training job	1 (4.2)
R1	1 (4.2)
R2	6 (25)
R3	5 (20.8)
**Prior hematology rotation type** (*n* = 26)	
Elective	5 (20.8)
Mandatory	19 (79.2)

The average duration of hematology rotation was 5.4 (SD: 1.8) weeks. The duration was 4 weeks in 37 (61.7%) of residents, 6 weeks in 4 (6.7%) and 8 weeks in 19 (31.7%). Table 2 shows the post-rotation satisfaction with teaching/training and knowledge gained on a 0 to 10 scale (0 = not satisfied at all and 10 = very satisfactory). Residents were overall satisfied with the explored items.

**Table 2:** Satisfaction of residents with teaching/training and knowledge gained during the hematology rotation

Item	Median (IQR)
Teaching/training by hematology staff	10 (10–10)
**Gaining knowledge**	
General hematology (anemia, hemoglobiopathies, bone marrow failure,.)	10 (9–10)
Hematological malignancies	10 (8–10)
Bleeding and thrombosis	10 (8–10)
Emergencies	9 (8–10)
**Usefulness in preparation for internal medicine board exam**	10 (9–10)

The pre- and post-rotation residents’ perception of the hematology career is shown in Table 3. There was a significant increase in all assessed perception items including considering hematology as a career.

**Table 3:** Internal medicine resident’s perception of hematology career pre- and post-hematology rotation

Item	Pre-rotation	Post-rotation	P value
	Median (IQR)		
**Consider hematology as a career** (0 = never, 10 = strongly agree)	7 (5–9)	8.5 (7–10)	0.0018
**Manageable workload** (0 = intolerable, 10 = very manageable)	8 (6–10)	10 (8–10)	< 0.0001
**Comfort in dealing with cancer patients** (0 = totally uncomfortable, 10 = very comfortable)	7 (4–9)	9 (7–10)	0.0005
**Perception of hematologist lifestyle** (0 = totally unsatisfactory, 10 = very satisfactory)	8 (6.5–9.5)	9 (7–10)	0.0203

The subgroup analysis of the change in considering hematology as a career following the hematology rotation is detailed in Table 4. The difference was statistically significant in all subgroups except in older (> 29 years) residents and those with prior hematology rotation or performing the rotation during the R1-R2 (junior) level.

**Table 4:** Subgroup analysis of Internal medicine resident’s perception of hematology career pre- and post-hematology rotation

Item	Pre-rotation	Post-rotation	P value
	Median (IQR)	Median (IQR)	
**Gender**			
Male	7 (5–8)	8 (6.5–9.5)	0.029
Female	8 (5.5–10)	10 (8.5–10)	0.016
**Age**			
> 29 years	8 (6-9.5)	8.5 (8–10)	0.1014
25to29 years	7 (5-8.5)	8.5 (6.5–10)	0.0068
**Center of internal medicine training**			
KSMC	7 (5-8.3)	8 (5.8–10)	0.0382
Other centers	7 (6–10)	9 (8–10)	0.0072
**Prior rotation**			
No	7 (4.5-8)	8 (7–10)	0.0018
Yes	8 (5–9)	9 (7–10)	0.3212
**Level of training**			
Junior (R1-R2)	6.5 (5–8)	8 (6–10)	0.2733
Senior (R3-R4)	8 (4–9)	9 (7–10)	0.0007

There was no significant difference in the change in considering hematology as a career according to the duration of the hematology rotation during which the study was conducted (Kruskal-Wallis test statistic = 0.1259, *p* = 0.937). Similarly, the correlation was not significant between the change in considering hematology as a career and the satisfaction of residents with teaching and knowledge gained during the hematology rotation. On the other hand, there was highly significant positive correlation between the change in considering hematology as a career and the change in the perception of workload manageability, comfort in dealing with cancer patients and hematologist lifestyle (Spearman’s rho = 0.404, 0.603 and 0.514; and *p* = 0.0014, < 0.0001 and < 0.0001; respectively).

## Discussion

To the best of our knowledge, this is the first study to explore the impact of a hematology rotation on the interest of internal medicine residents in considering a hematology career.

We found that hematology rotation is associated with a significant increase in interest in considering hematology as a career. Although no studies with a similar design exist for comparison, there is evidence supporting that hematology/oncology rotation is associated with pursuing a hematology-only career. In a study that included 626 hematology/oncology fellows in the United States, completing hematology/oncology rotations during internal medicine or pediatric clerkships was significantly (*p* = 0.01) positively associated with hematology-only career plans [15]. In the same study, fellows who had a hematology-only career plan were significantly (*p* < 0.01) more likely to report being encouraged to pursue a hematology career and having a clear vision of the hematology career path [15]. In another study, clinical experience during training and more exposure to role models/mentors had a significant effect on the choice of practicing non-malignant hematology among hematology-oncology fellows [16]. This further supports the findings of our study. On the other hand, in the study conducted by McFarland et al. [8], an inpatient hematology-oncology rotation was associated with a decrease in the interest of internal medicine residents in pursuing a hematology-oncology career. It should be noted that the later study was conducted in a single institution specialized in cancer care and the rotation was performed in a ward that admits oncology and malignant hematology patients, but not benign hematology.

Since the hematology rotation is already mandatory for internal medicine residents in Saudi Arabia, it is not possible to know the exact impact of this rotation on becoming hematologists. However, it is important to identify factors that may influence the attraction of more physicians to hematology careers. The number of variables explored in this study was limited; however, some of them correlated significantly with residents’ interests. The timing of performing the hematology rotation was one of these factors. There was no significant increase in interest among junior residents who were performing the rotation during the 1st two years of residency (R1-R2). While the increase in interest was highly significant among senior residents (R3-R4). Recent research found that specialties’ rotation schedule has an impact on career decisions among medical students [17]. In our setting, by the time senior residents (R3-R4) perform their rotations in hematology, they have already performed a good number of other subspecialty rotations that may have impacted their view about hematology. Another subgroup that did not show a significant increase in interest is the older (> 29 years) residents. These results suggest that performing the hematology rotation by younger residents during the 3rd -4th year of residency may increase the interest of residents in considering a hematology career.

There was no significant change in the interest of residents with prior hematology exposure in the current study. Those with prior rotation have already lived the experience of hematology rotation which may have resulted in a level of interest that did not change with further exposure.

Subspecialty rotations during internal medicine residency may be an opportunity to attract physicians to subspecialties with shortages. This is not limited to the hematology subspecialty as demonstrated in this study. A study that assessed the interest of internal medicine residents in a hepatology career before and after an inpatient hepatology rotation found a significant post-rotation increase in residents’ interest in hepatology [18].

A limitation of the study is that it is a single-institution one. Future studies including other training centers are needed to explore variables that may differ from one institution to another, such as workload. Another limitation is that it was not possible to know the impact of the change in interest on joining the hematology career because the questionnaire was anonymous. Also, factors found to impact residents’ attitudes towards considering a hematology career like mentorship and research experiences [11, 15], and other possibly relevant factors like comfort in dealing with benign hematology patients were not explored.

## Conclusion

In a single Saudi institution, hematology rotation was associated with a significant increase in the interest of internal medicine residents in considering a hematology career. Finding factors that may influence this interest in larger studies including other training centers is important to meet the needs for hematology specialists.

## Data Availability

The datasets used and/or analysed during the current study are available from the corresponding author on reasonable request.
